# Comprehensive Genomic Analysis and Expression Profiling of the C2H2 Zinc Finger Protein Family under Abiotic Stresses in Cucumber (*Cucumis sativus* L.)

**DOI:** 10.3390/genes11020171

**Published:** 2020-02-06

**Authors:** Yue Chen, Gang Wang, Jian Pan, Haifan Wen, Hui Du, Jingxian Sun, Keyan Zhang, Duo Lv, Huanle He, Run Cai, Junsong Pan

**Affiliations:** 1 School of Agriculture and Biology, Shanghai Jiao Tong University, Shanghai 200240, China; yuechen321@aliyun.com (Y.C.); wg@shu.edu.cn (G.W.); nillice@sjtu.edu.cn (J.P.); haifanwen@sjtu.edu.cn (H.W.); duhui1122@sjtu.edu.cn (H.D.); leyunu@sjtu.edu.cn (J.S.); lnykzky2016@sjtu.edu.cn (K.Z.); 18298345890@163.com (D.L.); hlhe75@sjtu.edu.cn (H.H.); cairun@sjtu.edu.cn (R.C.); 2 State Key Laboratory of Vegetable Germplasm Innovation, Tianjin 300384, China

**Keywords:** cucumber, C2H2 zinc finger protein, expression pattern, abiotic stress

## Abstract

Cucumber is one of the most important vegetables in the world. The C2H2 zinc finger protein (C2H2-ZFP) family plays an important role in the growth development and abiotic stress responses of plants. However, there have been no systematic studies on cucumber. In this study, we performed a genome-wide study of C2H2-ZFP genes and analyzed their chromosomal location, gene structure, conservation motif, and transcriptional expression. In total, 101 putative cucumber C2H2-ZFP genes were identified and divided into six groups (I–VI). RNA-seq transcriptome data on different organs revealed temporal and spatial expression specificity of the C2H2-ZFP genes. Expression analysis of sixteen selected C2H2-ZFP genes in response to cold, drought, salt, and abscisic acid (ABA) treatments by real-time quantitative polymerase chain reaction showed that C2H2-ZFP genes may be involved in different signaling pathways. These results provide valuable information for studying the function of cucumber C2H2-ZFP genes in the future.

## 1. Introduction

Transcription factors play an important role in plant growth development and abiotic stress responses. More than 60 transcription factor families have been reported in plants [1]. Among them, the zinc finger protein (ZFP) family is widely distributed in the genome of eukaryotes [2,3]. The first zinc finger transcription factor to be found was TFIIIA in *Xenopus oocytes* [4]. According to the number and position of Cys (cysteine) and His (histidine) residues, zinc finger proteins can be classified into C2H2, C4, C6, C4HC3, C3HC4, C2HC, C3H, and other types [5]. C2H2-ZFP, also known as the TFIIIA type, is currently the most widely distributed and well-studied class of ZFPs in eukaryotic genomes [6].

The first C2H2-ZFP gene found in plants was *EPF1* of *Petunia hybrid* [7]. Subsequently, many C2H2-ZFP genes were found in *Arabidopsis thaliana* (*Arabidopsis*) [8], rice [9], maize [10], potato [11], and other species [12,13]. Studies have shown that C2H2-ZFP genes comprise approximately 3%, 0.8%, and 0.7% of all genes in mammalia [6], yeast [14], and *Arabidopsis* [6], respectively.

C2H2-ZFP in eukaryotes generally has a specific conserved sequence consisting of 25–30 amino acids: XXCX (1-5)-CX (12)-HX (3-6)-H (where X represents any amino acid, and the parentheses indicate the number of amino acids). The finger contains two to three β-strands in its N-terminal sequence and one α-helix in the C-terminal [5]. Most plant C2H2-ZFPs generally have a highly conserved sequence of QALGGH, also called Q-type C2H2-ZFPs, which is absent from all animal zinc finger proteins [15]. In silico studies on ZFPs have identified 64 Q-type C2H2-ZFPs in *Arabidopsis* [6], 99 Q-type C2H2-ZFPs in rice [16], and 96 Q-type C2H2-ZFPs in durum wheat [17].

Recent studies have shown that C2H2-ZFPs play important roles in plant growth development and tolerance to adverse stress. A number of C2H2-ZFPs from rice and *Arabidopsis* are implicated in trichome initiation, seed germination, floral organogenesis, leaf initiation, and lateral shoot initiation [18,19,20,21,22,23]. C2H2-ZFPs are also involved in the following plant biotic and abiotic stresses: cold, salt, drought, and abscisic acid (ABA) stress in rice and *Arabidopsis* [24,25,26,27]; cold stress in banana [28]; cold and drought stress in soybean [29]; and biotic stress in potato [11]. These studies demonstrate that C2H2-ZFPs are involved in multiple growth processes and stress responses in plants.

Cucumber is one of the most important vegetables in the world. Due to the diversity of cucumber flower sexual types, it has become an ideal model for studying the sex differentiation mechanism [30,31]. Although C2H2-ZFPs are important, only one cucumber C2H2-ZFP has been reported to date [32]. Fortunately, the cucumber genome has been reported [33], which provides us with an opportunity to investigate C2H2-ZFPs.

In the present study, we identified 101 C2H2-ZFP genes from the cucumber genome and analyzed their chromosomal location, gene structure, conservation motif, and phylogenetic relationship. In addition, we also analyzed C2H2-ZFP gene expression profiles in different organs of cucumber. Some of them were selected as stress-related candidate genes to be tested under different abiotic conditions using real-time quantitative polymerase chain reaction (RT-qPCR). These results should provide valuable information for studying the function of cucumber C2H2-ZFPs in the future.

## 2. Materials and Methods

### 2.1. Identification of C2H2-ZFP Genes in Cucumber

Cucumber genome sequences were acquired from the Cucurbit Genomics Database (CuGenDB; [34]). The C2H2-ZFP sequences of *Arabidopsis* were obtained from the Arabidopsis Information Resource (TAIR; [35]). A two BLAST method was used to identify cucumber C2H2-ZFPs. First, *Arabidopsis* C2H2-ZFPs were used to search for possible cucumber C2H2-ZFPs with TBtools (e-value, 1 × 10^−5^) [36]. Second, all possible cucumber C2H2-ZFPs were further identified using National Center for Biotechnology Information (NCBI; [37]) BLASTP (e-value, 1 × 10^−5^). Finally, candidate proteins were confirmed with the SMART (http://smart.embl.de/) [38] and Pfam databases (http://pfam.xfam.org/) [39].

### 2.2. Chromosomal Location and Phylogenetic Analysis

Information on the distribution of cucumber C2H2-ZFP genes on the chromosomes was obtained using the TBtools software (GitHub, San Francisco, CA, USA). Cucumber and *Arabidopsis* C2H2-ZFP protein sequences were used for phylogenetic analysis. The MEGAX software was used to construct a phylogenetic tree using the maximum likelihood (ML) method with partial deletion of 1000 bootstraps and a WAG model. The results were formatted for display using Evolview V3 [40,41].

### 2.3. Collinearity Analysis

Gene duplication analysis of Trihelix genes in different species was performed using the Multiple Collinearity Scan software toolkit (MCScanX) (Plant Genome Mapping Laboratory, University of Georgia, Athens, GA, USA) with default parameters [42]. We plotted the collinearity relationship of the C2H2-ZFP genes from selected species using the TBtools software.

### 2.4. Gene Structure and Motif Analysis

The gene structure of cucumber C2H2-ZFP genes was identified via TBtools. The online Multiple Expectation Maximization for Motif Elicitation (MEME) program version 5.0.5 [43] was used to identify conserved motifs of the cucumber C2H2-ZFPs [44].

### 2.5. Expression Profile Analysis

To analyze the expression profiles of cucumber C2H2-ZFP genes in different organs, we retrieved public RNA-seq data (accession number: SRP071224) from the National Center for Biotechnology Information’s Short Read Archive database. Analysis of RNA-Seq data from 23 sampled cucumber tissues was performed based on the regular protocol by Yang et al. [45]. The heatmap of cucumber C2H2-ZFP gene expression profiles was generated using the TBtools software.

### 2.6. Expression Analysis of Abiotic-Stress-Responsive C2H2-ZFP Genes in Cucumber

Cucumbers from the inbred line 9930 were used as plant materials. The experiment was implemented at Shanghai Jiao Tong University (Shanghai, China). Plants were grown in a growth room at day/night temperatures of 24/18 °C with a 16 h photoperiod. When the cucumber seedlings were at the five-true leaf stage, we conducted the following treatments: 100 mM NaCl, 100 g/L Polyethylene glycol (PEG) 6000, 100 μM ABA, and 4 °C. The NaCl and PEG6000 were added into a nutrient solution and hormones were sprayed onto the leaves. For cold treatment, cucumber seedlings were moved into an illuminated incubator. Leaves from three different plants were harvested at 0, 1, 3, 6, 12, and 24 h after treatment. Each experiment was repeated at least three times. All samples were frozen with liquid N_2_ and stored at −80 °C for RNA isolation.

Total RNA was isolated from leaves using an OminiPlant RNA Kit (CWBIO, Beijing, China). Complementary DNA (cDNA) was synthesized from 1 μg of total RNA using a HiFiScript cDNA Synthesis Kit (CWBIO, Beijing, China). Quantitative real-time PCR was carried out using the UltraSYBR Mixture (CWBIO, Beijing, China) PCR reactions were prepared and performed according to the manufacturers’ protocol. The *β-actin* gene (GenBank: AB010922) of cucumber was used as an internal control. Geneious software was used to design primers according to the cDNA sequences (Table S1). PCR-amplified product lengths were 100–150 bp. The analysis of relative microRNA (mRNA) expression data was performed using the 2^−ΔΔ*C*t^ method [46]. Each expression profile was independently verified in three replicate experiments performed under identical conditions.

## 3. Results

### 3.1. Identification of C2H2-ZFP Genes in Cucumber

A two BLAST method was used to identify cucumber C2H2-ZFPs using sequences of *Arabidopsis* C2H2-ZFPs as a query sequence. We found 105 candidate genes, and all putative genes were subsequently verified in the SMART and Pfam databases to confirm the existence of the C2H2-ZFP domains. In total, 101 C2H2-ZFP genes were detected and verified.

The basic information for the 101 C2H2-ZFP genes of cucumber is provided in Table S2, including coding sequence (CDS), amino acid sequence, isoelectric point (pI), and molecular weight (MW). Csa5G477610 was the smallest protein with 103 amino acids, whereas Csa6G487810 was the largest protein with 1464 amino acids. The protein MWs varied from 11.31 kDa (Csa1G657510) to 164.89 kDa (Csa6G487810), and the pIs ranged from 4.97 (Csa6G509590) to 9.96 (Csa6G312040).

### 3.2. Chromosomal Distributions and Phylogenetic Analysis

**Figure 1:**
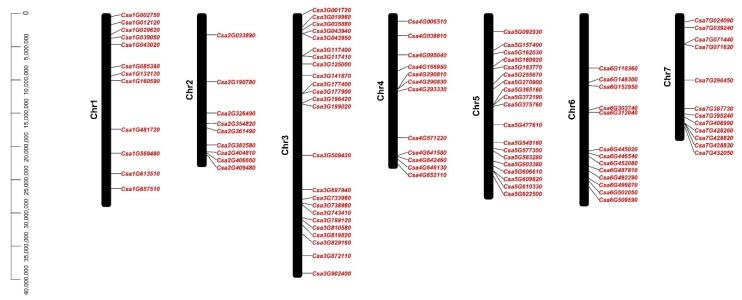
The gene locations of C2H2 zinc finger protein (C2H2-ZFP) genes in cucumber. The scale bar on the left represents the length of the chromosome (bp).

The 101 C2H2-ZFP genes are distributed across seven cucumber chromosomes, ranging from 9 to 24 genes per chromosome (Figure 1). Chromosome (Chr) 3, the longest chromosome, also contained the highest number of C2H2-ZFP genes. Chr2 was longer than Chr7 but contained the least number of C2H2-ZFP genes.

**Figure 2:**
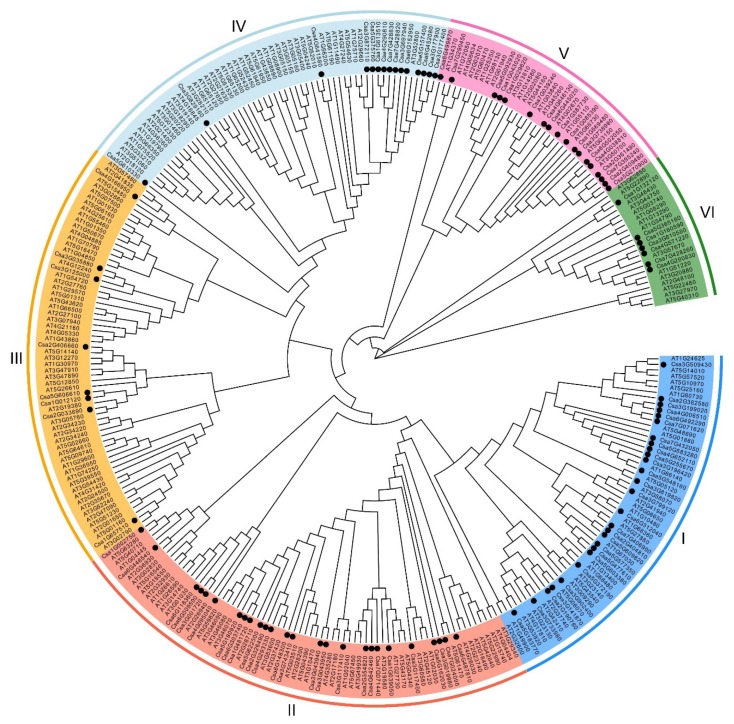
Phylogenetic analysis of C2H2-ZFPs in *Arabidopsis* and cucumber. The phylogenetic tree was based on C2H2-ZFP sequences from cucumber (101 proteins, marked with a black dot) and *Arabidopsis* (208 proteins). Each of the six C2H2-ZFP groups is indicated in a specific color.

To investigate the evolutionary relationship of C2H2-ZFP genes, a phylogenetic tree was constructed using full-length C2H2-ZFP protein sequences of cucumber and *Arabidopsis* (Figure 2). The C2H2-ZFPs from both species were divided into six groups named I to VI. Groups I and II were the biggest groups and included 27 and 26 members, respectively. The smallest group (Group VI) had seven members from cucumber.

### 3.3. Collinearity Analysis of the Relationship among Cucumber, Melon(Cucumis melo), and Arabidopsis Members

**Figure 3:**
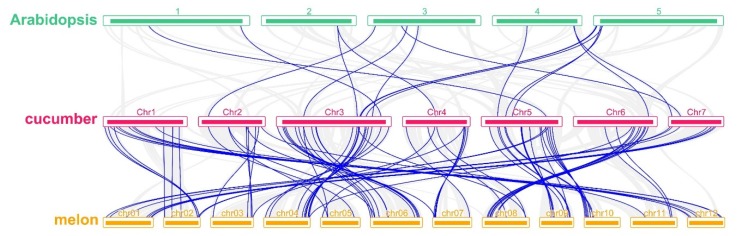
Collinear relationships of C2H2-ZFPs among cucumber, melon (*Cucumis melo*), and *Arabidopsis*. Gray lines in the background indicate collinear blocks within cucumber, melon, and *Arabidopsis* genomes while blue lines highlight the collinear C2H2-ZFP gene pairs.

We performed a comparative analysis to identify the collinear C2H2-ZFP genes among melon, cucumber, and *Arabidopsis* (Figure 3). Only 15 genes from cucumber shared collinear relationships with *Arabidopsis*. C2H2-ZFP genes on cucumber chromosome 1 showed no collinearity with *Arabidopsis*. However, the collinear relationships of C2H2-ZFP genes between cucumber and melon were quite rich. In total, 79 C2H2-ZFP genes in cucumber were collinear in melon (Table S3). These results suggest that C2H2-ZFP genes in cucumber and melon have strong relationships.

### 3.4. C2H2-ZFP Gene Structures and Conserved Motifs

**Figure 4:**
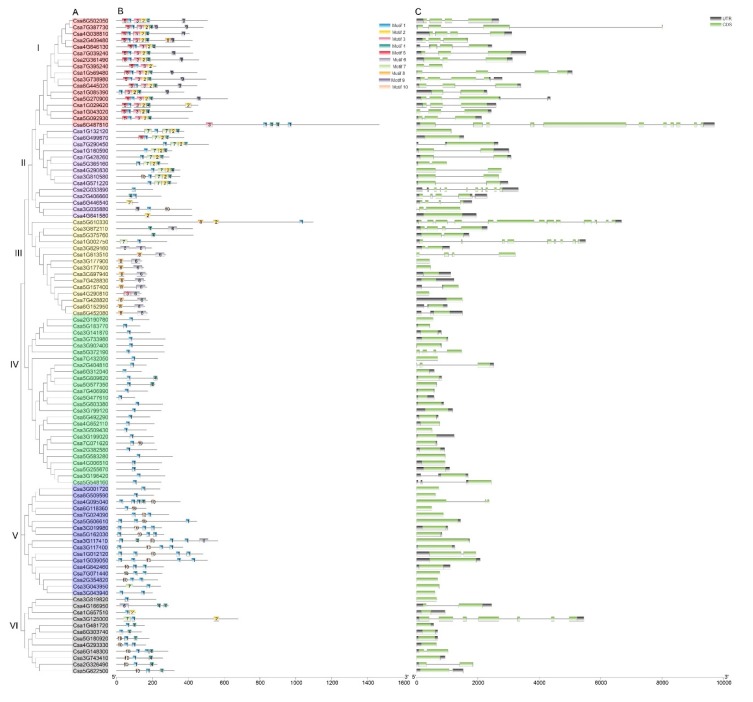
Phylogenetic tree, motif analysis, and gene structure of cucumber C2H2-ZFP genes. (**A**). Phylogenetic analysis of 101 cucumber C2H2-ZFPs was conducted using the MEGA X program. Each of the six C2H2-ZFP groups is indicated in a specific color. (**B**). Conserved motif analysis of C2H2-ZFPs was conducted using MEME tools. Conserved motifs are shown in different colored boxes. (**C**). Gene structure analysis of C2H2-ZFP genes. Coding sequences (CDS) and untranslated regions (UTRs) are represented by black and green boxes, respectively. Introns are represented by black lines.

To better understand the phylogenetic relationships between the structure and function among cucumber C2H2-ZFP genes, the conserved motifs and exon/intron structure were analyzed (Figure 4). In the phylogenetic tree, all 101 C2H2-ZFP genes were divided into six groups, namely I, II, III, IV, V, and VI, containing 17, 14, 15, 26, 17, and 22 members, respectively. We identified ten conserved motifs among the 101 C2H2-ZFPs (Table S4). Domain 1 includes a plant-specific conserved sequence (‘QALGGH’), which was present in 85 proteins. In addition, the most closely related members had a common motif composition. For example, motifs 1, 2, 3, 4, and 5 were observed in most group I members. Motifs 6 and 8 were specifically detected in group III. The great majority of group IV members only contained motif 1.

Analysis of the structure of C2H2-ZFP genes also produced a similar result. C2H2-ZFP genes in the same group shared similar numbers of exons/introns. For example, most members in groups IV and V had one exon, while all members in group I had more than two exons.

### 3.5. Expression Profiles of Cucumber C2H2-ZFP Genes in Different Tissues

**Figure 5:**
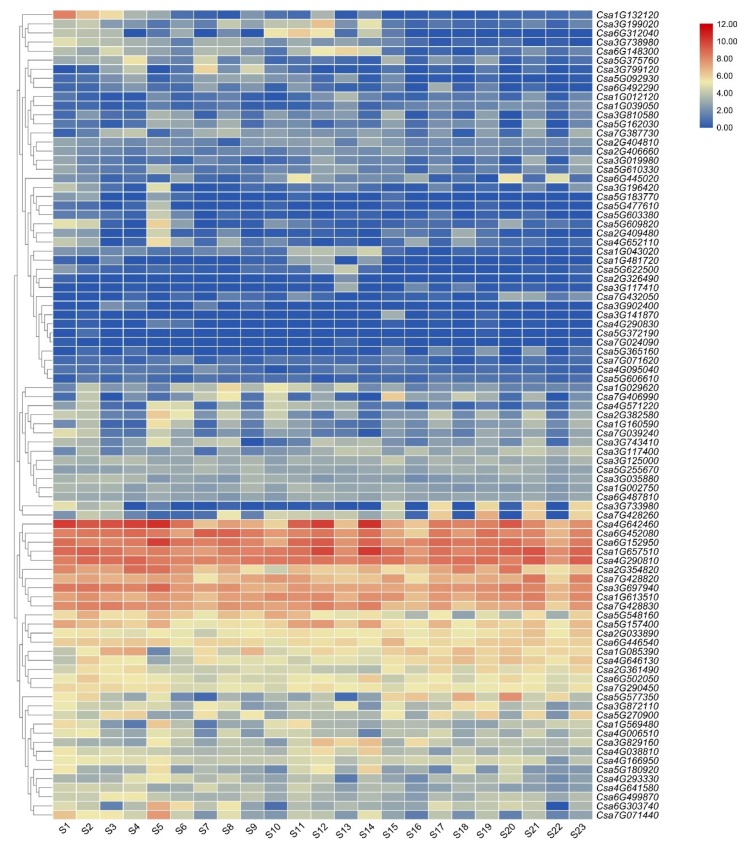
A heatmap showing the expression of C2H2-ZFP genes in different tissues. The following abbreviations are used: S1, roots of 4-week-old seedlings; S2, hypocotyl of 4-week-old seedlings; S3, cotyledon of 4-week-old seedlings; S4, true leaf of 4-week-old seedlings; S5, root; S6, stem; S7, young leaf (the second node from the top of cucumber plants); S8, petiole of young leaf; S9, old leaf (the fifth node from the top of cucumber plants); S10, petiole of old leaf; S11, tendril; S12, female flower; S13, male flower bud; S14, male flower; S15, unfertilized ovary; S16, peel of unfertilized ovary; S17, flesh of unfertilized ovary; S18, peel of 1-week-old fruit; S19, flesh of 1-week-old fruit; S20, peel of 2-week-old fruit; S21, flesh of 2-week-old fruit; S22, peel of 3-week-old fruit; S23, flesh of 3-week-old fruit. [45]. Genes highly expressed in tissues are colored red and genes not expressed in tissues are colored blue.

To analyze the tissue specificity in cucumber, a heatmap of 89 cucumber C2H2-ZFP gene expression profiles in 23 cucumber tissues was generated (Figure 5, Table S5).

The results showed that the C2H2-ZFP genes presented diverse expression profiles among different tissues, implying distinct roles for various developmental stages. The gene expression levels including *Csa2G354820*, *Csa7G428820*, *Csa3G697940*, *Csa1G613510*, *Csa7G428830*, *Csa4G642460*, *Csa6G452080*, *Csa6G152950*, *Csa1G657150*, and *Csa4G290810* were consistently high in all organs. In contrast, *Csa3G902400*, *Csa3G141870*, *Csa4G290830*, *Csa5G372190*, *Csa7G024090*, *Csa5G365160*, *Csa7G071620*, *Csa4G095040*, and *Csa5G606610* were barely expressed in all organs. In addition, several genes were observed in specific tissues as follows: *Csa1G132120* showed specific expression in roots and hypocotyl; and *Csa3G199020* had the strongest expression in flowers. These genes may be involved in the development of the corresponding phenotype.

### 3.6. Expression Analysis of C2H2-ZFP Genes under Abiotic Stress

**Figure 6:**
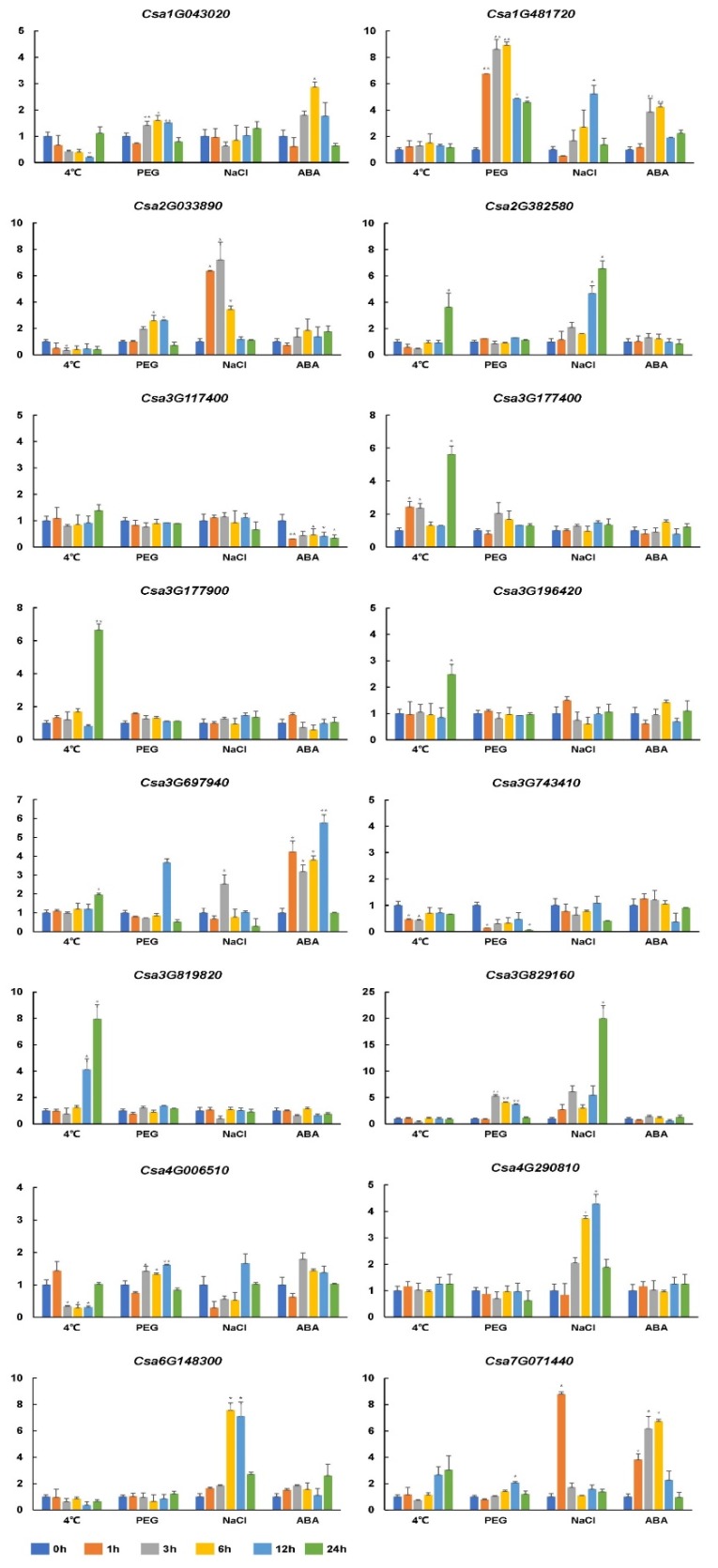
Expression analysis of 16 selected C2H2-ZFP genes in leaf tissues under cold, drought, salt, and abscisic acid (ABA) stresses using qRT-PCR. The relative microRNA (mRNA) abundance of 16 selected C2HC2-ZFP genes was normalized with respect to the reference β-actin gene (GenBank AB010922). The *x*-axis represents time points after stress treatments. The *y*-axis is the scale of the relative transcript abundance level. Error bars represent the standard deviations from three biological replicates. Asterisks indicate stress treatment groups that showed a significant difference in transcript abundance compared to the control group (* *P* < 0.05 and ** *P* < 0.01).

To demonstrate if cucumber C2H2-ZFP genes are involved in stress tolerance or response to hormones, 16 cucumber homologous genes of *Arabidopsis* abiotic-stress-responsive genes were selected. The expression patterns of 16 C2H2-ZFP genes in leaf tissues were analyzed under low temperature (4 °C), drought (PEG), NaCl, and ABA treatments. As shown in Figure 6, all sixteen C2H2-ZFP genes were induced by at least one treatment.

Under cold stress treatment, ten genes were induced. The transcription of four genes (*Csa1G043020*, *Csa2G033890*, *Csa3G743410*, and *Csa4G006510*) was inhibited, whereas the transcript level of the other six genes was increased. The six upregulated genes were induced significantly at 24 h after cold treatment. Eight genes were mediated by PEG treatment as follows: seven genes were upregulated, and one gene (*Csa3G743410*) was downregulated. The same eight genes were upregulated after NaCl treatment. The transcript of *Csa3G829160* was strongly upregulated (>20-fold) at 6 h after NaCl treatment. Only five genes were mediated by ABA treatment as follows: four genes were upregulated, and one gene (*Csa3G117400*) was downregulated.

Among the 16 C2H2-ZFP genes, only one gene (*Csa3G697940*) responded to all four treatments, and two genes (*Csa2G033890* and *Csa2G382580*) responded to three treatments. The remaining thirteen genes were mediated by one or two treatments. The expression profile indicated that C2H2-ZFP genes may be involved in cucumber responses to abiotic stress, especially to low temperature, high temperature, and NaCl treatment.

## 4. Discussion

C2H2-ZFPs mainly participate in growth development and abiotic stress responses of plants as thoroughly studied in rice [16], durum wheat [17], and poplar plants [15,47]. However few studies have investigated C2H2-ZFP genes in cucumber. In this study, we identified a total of 101 cucumber C2H2-ZFP genes. The C2H2-ZFP gene family has 176 members in *Arabidopsis*. The genome of cucumber (genome size of 367 Mb) is larger than the genome of *Arabidopsis* (genome size of 125 Mb) [33], but the number of ZFPs identified in this study is less in cucumber than in *Arabidopsis*. Whole-genome duplication (WGD) is common in angiosperm plants and produces a tremendous source of raw material for gene genesis. Previous research has revealed three WGD events in *Arabidopsis*. A paleohexaploidy (γ) event occurred in *Arabidopsis* after the divergence of monocotyledons and dicotyledons. Subsequently, two WGDs (α and β) occurred in *Arabidopsis.* The recent WGD events have played an important role in the rapid expansion of gene families [48]. However, cucumber lacks recent WGD events [33,49], resulting in the low number of cucumber C2H2-ZFP genes.

The collinearity analysis revealed that there are only 16 pairs of homologous genes in cucumber and *Arabidopsis* but that more orthologous gene pairs are present in cucumber and melon (Figure 3), which is consistent with the phylogenetic relationship among cucumber, melon, and *Arabidopsis*. According to the number of melon and cucumber homologous genes, we found that cucumber chromosomes 1, 2, 3, 5, and 6 were collinear to melon chromosomes 2/12, 3/5, 4/6, 9/10, and 8/11, respectively, indicating that these cucumber chromosomes each resulted from a fusion of two ancestral chromosomes after speciation [33].

To analyze the evolutionary relationship of cucumber C2H2-ZFPs, a phylogenetic tree was constructed. In Figure 2, we found cucumber’s *Tu* (*Csa5G577350*) gene to be closely associated with *Arabidopsis ATZFP6* (*AT1G67030*). *Tu* is implicated in cucumber fruit tuberculate development [32]. *AtZFP6* plays a key role in regulating trichome development [50]. This result revealed that C2H2 genes may have conserved functions in the same group.

Generally, C2H2-ZFPs with a similar number of exons and motif composition were classified in the same groups (Figure 4). The domains and motifs of transcription factors are often related to protein interaction, transcriptional activity, and DNA binding [51]. The motif analysis showed that most of the C2H2-ZFPs of cucumber contained the Q-type motif 1, ‘QALGGH’, plant-specific sequence. In our study, approximately 84% (85 out of 101) of the cucumber C2H2-ZFPs belonged to Q-type C2H2-ZFPs, which is a large number compared to that of *Arabidopsis* (34%, 64 out of 176) [6] and rice (52%, 99 out of 189) [16], but similar to that of durum wheat (79%, 96 out of 122) [17]. Studies have shown that residues of the Q-type motif play an important role in binding activity [52]. Motifs 6 and 8 were specifically detected in group III, indicating that they play an important role in this subfamily. These results suggest that although some motifs of the C2H2-ZFPs are highly conserved, the new evolutionary motifs may perform new functions in some plants and that the function of these motifs requires further verification.

C2H2-ZFP genes play a role in specific tissues at certain stages of plant growth development. Transcriptome data from different tissues and periods were used to analyze the expression profiles of cucumber C2H2-ZFPs. *Csa6G303740* had the strongest expression in roots. *ZAT11*, a homologous gene of *Csa6G303740*, was highly expressed in roots and particularly in root tips [53]. *Csa7G428260* transcript signals were detected only in flesh. *MaC2H2-1/2* (two C2H2-ZFPs) is involved in the regulation of fruit ripening in banana via transcriptional repression of ethylene biosynthetic genes [28]. These results may reveal the similar biological roles of these genes in different species. *Csa3G199020* showed specific expression in flowers. *AtZFP1*, an *Arabidopsis* ortholog of *Csa3G199020*, is highly expressed in the shoot apex and plays an important role in shoot development [20]. These results suggest that their functions may vary in different plant species.

*Arabidopsis* Transgenic *ATHB7::* GUS plants showed no or very low GUS activity in all tissues under optimal water conditions. However, the GUS activity was obviously induced in plants subjected to a limited water supply [54]. *ATHB12*, a paralogous gene of *ATHB7*, has a similar expression pattern and function [55]. Some genes were expressed at high levels in each tissue throughout the cucumber developmental stages, which suggests that these genes may be involved in the entire life cycle of cucumber. Although some genes were rarely expressed in all tissues, it did not indicate that they had no function in cucumber. It is possible that these genes may be expressed under specific environmental conditions.

Many studies have shown that C2H2-ZFP genes respond to abiotic stress and their expression changes after different time points after stress treatment [15,47,56]. In our study, 16 genes were induced by at least one type of stress. Ten genes were induced by cold treatment, indicating that cucumber C2H2-ZFP genes may be more sensitive to low temperatures. *Csa3G697940* was induced by all treatments. Correspondingly, *Arabidopsis AtSAP5*, a homologous *Csa3G697940* gene, also responded to environmental challenges, including salt, osmotic, cold, and ABA stress [8]. Previous studies have shown that osmotic stress response is highly related to ABA response [56]. In our study, five genes were induced by ABA stress, three of which also responded to NaCl stress. These results demonstrate that zinc finger proteins may be involved in multiple environmental stresses and are worthy of further investigation in the future.

## 5. Conclusions

In this study, 101 cucumber C2H2-ZFP genes were identified and divided into six groups (I–VI). Chromosomal location revealed that the 101 cucumber C2H2-ZFP genes were distributed in all cucumber chromosomes. C2H2-ZFPs with a similar number of exons and motif composition were classified in the same groups. RNA-seq transcriptome data of different organs revealed temporal and spatial expression specificity of the C2H2-ZFP genes. Expression analysis of sixteen selected C2H2-ZFP genes in response to cold, drought, salt, and ABA treatments by RT-qPCR showed that C2H2-ZFP genes may be involved in different signaling pathways. These results provide valuable information for studying the function of cucumber C2H2-ZFP genes in the future.

## Supplementary Materials

The following are available online at https://www.mdpi.com/2073-4425/11/2/171/s1. Table S1: Primers for RT-qPCR analysis of selected C2H2-ZFP genes in cucumber, Table S2: The basic information on 101 C2H2-ZFP genes, Table S3: Collinear relationships of C2H2-ZFP family members in cucumber and between melon and *Arabidopsis*, Table S4: Conserved motif analysis of C2H2-ZFPs using MEME tools. Table S5: Expression levels of cucumber C2H2-ZFP genes in different tissues.
